# Association of Exercise Capacity, Cardiac Function, and Coronary Artery Calcification with Components for Metabolic Syndrome

**DOI:** 10.1155/2018/4619867

**Published:** 2018-10-04

**Authors:** Hyun Jun Kim, Ji Hee Kim, Min Cheol Joo

**Affiliations:** Department of Rehabilitation Medicine and Institute of Wonkwang Medical Science, Wonkwang University School of Medicine, Iksan, Republic of Korea

## Abstract

**Purpose:**

The purpose of this study was to investigate the association of exercise capacity, cardiac function, and coronary artery calcification (CAC) with components of metabolic syndrome in Korean adults.

**Method:**

Medical records of healthy adults who underwent exercise tolerance test (ETT), coronary CT angiography (CTA), and echocardiography of the heart for cardiac health check-up were retrospectively reviewed. Patients who had a history of severe cardiovascular disease or could not perform ETT due to other musculoskeletal problems were excluded. Subjects were classified into groups based on the number of components for metabolic syndrome: no component (Group 1, n=90), 1, 2 components (Group 2, n=321), and 3 or more components (Group 3, n=154). Exercise capacity was assessed using the symptom-limited ETT, and CAC score was obtained using the coronary CTA and Agatston score. Cardiac structure and function were assessed using echocardiography.

**Results:**

A total of 565 patients (mean (SD) age 59.5 (9.1), 340 men, 225 women) were selected. Exercise capacity was significantly lower in Group 3 than in the other groups (p<0.05). The CAC score was significantly higher in Group 3 than in the other groups (p<0.05). Compared to the other groups, echocardiography findings in Group 3 showed a greater hypertrophy of the left ventricle and reduction in the diastolic function (p<0.05). Exercise capacity, CAC score, cardiac structure, and function were different between the 3 groups, where a tendency to worsen was observed from Group 1 to Group 3.

**Conclusion:**

Metabolic syndrome decreases exercise capacity of the patient and contributes to CAC, thereby increasing the risk for cardiovascular diseases and deterioration in cardiac structure and function. Therefore, early detection of metabolic syndrome and subsequently the prevention and management of heart disease are necessary.

## 1. Introduction

It has been reported that metabolic syndrome increases the mortality rate due to coronary artery disease (CAD) by 2.9-4.2 times and that due to cardiovascular disease by 2.6-3.0 times [1]. In a previous study that analyzed the relationship between metabolic syndrome and CAD using coronary CT angiography (CTA), a tendency towards increased coronary artery calcification (CAC) with an increase in the number of components for metabolic syndrome was seen [2]. This indicated that patients with metabolic syndrome have a higher risk of developing cardiovascular diseases [3]. The echocardiography of patients with metabolic syndrome showed deterioration in the cardiac structure and function [4–6]; this is known to further reduce the exercise capacity [7]. In a study in which an exercise tolerance test (ETT) was conducted in patients with metabolic syndrome, a lower exercise duration and maximum oxygen intake was reported [8], while another study reported that metabolic syndrome in patients with CAD is associated with reduced exercise capacity and heart rate recovery (HRR) [9, 10].

Metabolic syndrome is known to affect exercise capacity, and cardiac structure and function, and cause CAC. In Korea, however, there have been very few studies about the association of exercise capacity, and cardiac structure and function with the components for metabolic syndrome [11]. Furthermore, most previous studies used the NCEP-ATP III criteria [12], which are used for the diagnosis of metabolic syndrome. The NCEP-ATP criteria were established based on Caucasian study subjects. Asians, including Koreans, have a relatively lower rate of obesity compared to Caucasians [13]. It is necessary to use criteria that are more appropriate for Koreans, rather than the aforementioned criteria of using the waist circumference for the diagnosis of metabolic syndrome [14, 15]. In addition, there is a lack of studies about the association of exercise capacity, CAC, and cardiac structure and function, with each component.

The purpose of this study was to use the ATP III-BMI 25 criteria [16], which are used for the diagnosis of metabolic syndrome among Asians, in Korean adults, to analyze the association of exercise capacity, CAC, and cardiac structure and function with the components for metabolic syndrome, and the correlations between them.

## 2. Materials and Methods

### 2.1. Materials

**Figure 1:**
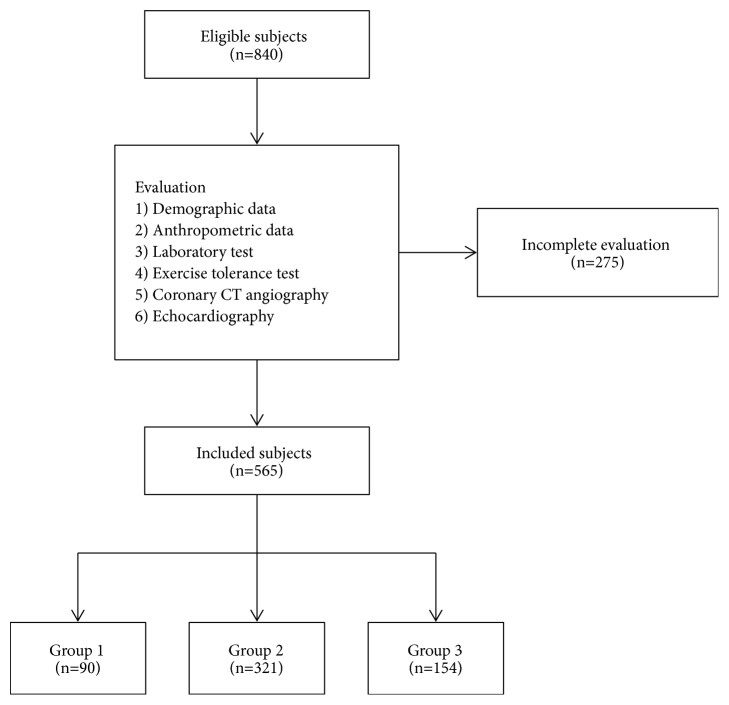
Flow chart for this study.

Medical records of 840 healthy adults, at least 20 years of age, who underwent a cardiac health check-up at Wonkwang University Hospital from September 2012 to March 2016, were retrospectively reviewed. Finally, 565 subjects who was finished all of the anthropometric data, laboratory tests, ETT, coronary CTA, and echocardiography were enrolled. (Figure 1) Based on the number of components for metabolic syndrome, the patients were assigned to different groups: no component (Group 1), 1, 2 components (Group 2), and 3 or more components (Group 3). The diagnosis of metabolic syndrome was based on the NCEP-ATP III criteria, and modified ATP III-BMI 25 criteria that convert the waist circumference to body mass index (BMI) (Table 1).

Patients who had a history of severe cardiovascular disease or could not perform ETT due to other musculoskeletal problems were excluded. Severe cardiovascular disease included a history of stable or unstable angina pectoris, myocardial infarction or heart failure.

This study was approved by the Institutional Review Board (IRB) of Wonkwang University Hospital (IRB Number: 201604-hre-037) and complied with all the necessary criteria.

#### 2.1.1. Anthropometric Measurement and Laboratory Test

Anthropometric data were obtained using a body composition analyzer (Ioi353, Jawon medical, Gyeongsan, South Korea), and the BMI was calculated as weight (kg)/height^2^ (m^2^). Laboratory tests were performed after 8 hours of fasting and included HbA1c, fasting blood glucose, and lipid battery.

#### 2.1.2. Exercise Tolerance Test

A Treadmill designed for ETT (Q-stress TM55, Mortara Instrument, Milwaukee, USA) was used to conduct symptom-limited graded maximum exercise test based on the Bruce protocol. The test used a real-time recording of a 12-lead electrocardiogram (Quinton Q-stress, Mortara Instrument, Milwaukee, USA), automated blood pressure, and pulse monitor (247BP, SunTech Medical, Morrisville, USA).

During the ETT, resting and maximal heart rate (HR), resting and maximal systolic and diastolic blood pressure (SBP and DBP), and exercise duration were measured. During maximum exercise, the metabolic equivalent (MET) was calculated using the speed and incline of the stage of maximum possible exercise. HRR after exercise was calculated using the value obtained by subtracting the HR at one minute during recovery, from the maximum HR during the exercise [17].

#### 2.1.3. Coronary Artery Calcification

In order to measure the calcification of the coronary arteries, a 256-channel computed tomography (CT) (Somatom Definition Flash, Siemens Medical Solution, Forchheim, Germany) was used to perform coronary CTA at 3 mm thickness and 120 kV, 370 mA. To determine the level of calcification, coronary calcium quantification software was used and Agatston score method [18] was applied to calculate the CAC score. The scores of the left main artery (LMA), left anterior descending artery (LADA), left circumflex artery (LCXA), right coronary artery (RCA), and the total score, as well as calcium volume, were measured.

#### 2.1.4. Echocardiography

Echocardiography was performed to assess the cardiac structure and functions. An experienced expert used Vivid E95 (GE Healthcare, Chicago, USA), EPIQ 7 (Philips, Andover, Massachusetts, USA), and IE 33 (Philips, Andover, USA) to test the subjects in a left lateral decubitus position. Echocardiography was performed following the standardized method suggested by the American Echocardiography Society [19], to obtain the left ventricular end-diastolic diameter (LVEDD), left ventricular end-systolic diameter, (LVESD), interventricular septum (IVS) thickness, posterior wall (PW) thickness, left ventricular ejection fraction (LVEF), peak early diastolic transmitral flow (E), peak late diastolic transmitral flow (A), and E/A ratio.

#### 2.1.5. Statistical Analysis

SPSS 18.0 (IBM Corp., Armonk, USA) was used for statistical analysis. In order to compare the age, BMI, laboratory test results, ETT results, CAC score, and echocardiography results of the 3 groups, one-way ANOVA was performed. Logistic regression analysis accounting for demographics and clinical variables were performed. If a statistically significant difference was found, Bonferoni method was applied for the post hoc test, and if an equal variance was not found, Tamhane's T2 method was applied. To identify the correlation between the results of ETT, CAC, and echocardiography, Pearson correlation coefficient was performed. All statistical significance was based on a p value less than 0.05.

## 3. Results

### 3.1. Patient Characteristics and Laboratory Test

Among the 565 subjects, the mean (SD) age was 59.5(9.1) years. Number and percentage of subjects that have each components of metabolic syndrome are as shown at Table 2. The number of subjects in the groups was Group 1 = 90, Group 2 = 321, and Group 3 = 154. There was no significant difference in terms of sex, age, and height between the groups (Table 3). Weight and BMI appeared to increase from Group 1 to Group 3 (p<0.05). On laboratory test, HbA1c, fasting blood glucose, and triglycerides increased from Group 1 to Group 3 (p<0.05), while HDL-C decreased from Group 1 to Group 3 (p<0.05).

#### 3.1.1. Exercise Tolerance Test

The exercise capacity, exercise duration, and METs during maximal exercise were significantly lower in Group 3 than in Group 1 (p<0.05) (Table 4). HRR after exercise was significantly lower in Group 3 than in Group 1 and Group 2 (p<0.05).

#### 3.1.2. Coronary Artery Calcification

There was no difference in the CAC score of LMA between the three groups, while that of the LADA was significantly higher in Group 3 than in Group 1 and Group 2 (p<0.05). CAC scores of LCXA and RCA were significantly higher in Group 2 and Group 3 than in Group 1 (p<0.05). The sum of the CAC scores and the calcium volume increased from Group 1 to Group 3 (p<0.05) (Table 4).

#### 3.1.3. Echocardiography

LVEDD was significantly thicker in Group 3 than in Group 1 (p<0.05), and there was no difference in the LVESD between the three groups. The thickness of IVS and PW increased as they progressed to Group 3 (p<0.05). No difference was found in the LVEF and peak early diastolic transmitral flow (E) between the groups. Peak late diastolic transmitral flow (A) and E/A ratio were significantly higher in Group 2 and Group 3 than in Group 1 (p<0.05) (Table 4).

#### 3.1.4. Results of Multivariate Logistic Regression Analysis

In the correlation analysis between ETT, Agatston score and echocardiography showed Table 5. Odds ratio of total Agatston score and calcium volume showed 3.003 between Groups 1 and 3.

#### 3.1.5. Correlation between Exercise Tolerance Test and Coronary Artery Calcification

In the correlation analysis between exercise capacity and CAC score, exercise duration showed a significant negative correlation with the CAC score of RCA, total calcium score, and calcium volume (p<0.05) (Table 6). METs during maximal exercise and HRR did not show a statistically significant correlation with the CAC score of any coronary artery, total calcium score, or with calcium volume.

#### 3.1.6. Correlation between Exercise Tolerance Test and Echocardiography Results

In the correlation analysis between ETT and echocardiography, IVS and PW thickness showed a significant negative correlation with HRR after exercise (p<0.05) (Table 7). Peak early diastolic transmitral flow (E) and METs during maximal exercise showed a significant negative correlation (p<0.05). Peak late diastolic transmitral flow (A) and METs during maximal exercise and exercise duration showed a significant negative correlation (p<0.05). E/A ratio and exercise duration, and HRR showed a significant positive correlation (p<0.05).

#### 3.1.7. Correlation between Coronary Artery Calcification and Echocardiography Result

In the correlation analysis between the CAC score and echocardiography, IVS and PW, the PW thickness showed a significant positive correlation with the CAC score of LADA (p<0.05) (Table 8). Peak late diastolic transmitral flow (A), LMA, LADA, and LCXA showed a significant correlation with the total CAC score and calcium volume (p<0.05). LVEDD, LVESD, LVEF, and peak early diastolic transmitral flow (E) did not show a significant correlation with the CAC score.

## 4. Discussion

In this study, healthy adults who were admitted for cardiac health check-up population were classified into 3 groups, based on the number of components for metabolic syndrome, to compare their exercise capacity and CAC score using coronary CTA, as well as to compare the cardiac structure and function using echocardiography. The correlation between exercise capacity and CAC score, and the cardiac structure and function were also investigated.

The NCEP-ATP III, currently a widely used definition for metabolic syndrome and diagnosis, defines metabolic syndrome as a presence of 3 or more parameters from the list of criteria. In 2004 however, new criteria were defined for Asians by the WHO's Asia-Pacific region. Obesity was defined as a BMI of 25 kg/m^2^ and above. People whose waist circumference is over 90 cm in men and 80 cm in women were considered to be at a high risk for metabolic syndrome [20]. Shiwaku et al. [16] claimed that though obesity is less common in Asians than in Caucasians, metabolic diseases related to obesity were more common at a lower BMI in Asians compared to Caucasians, and thus, different criteria for the definition and diagnosis of metabolic syndrome need to be considered based on ethnicity.

To determine the appropriate criteria for the diagnosis of metabolic syndrome in Asians, a cross-sectional study including the Japanese, Koreans, and Mongolians was conducted. Among the diagnostic criteria for metabolic syndrome, the diagnosis sensitivity increased when BMI over 25 kg/m^2^ was used instead of waist circumference, and ATP III-BMI 25 criteria were suggested [16].

Therefore, our study analyzed the effects of metabolic syndrome on exercise capacity, CAC score, and cardiac structure and function by using the ATP III-BMI 25 criteria, which are appropriate for Koreans.

In this study, METs during maximal exercise, exercise capacity scale, exercise duration, and HRR were all significantly lower in Group 3. The decrease in HRR is a risk factor for mortality due to cardiovascular diseases [17]. Old patients with CAD having metabolic syndrome have been associated with low HRR [9, 21]. The results of this study are in line with the results of previous studies [9, 22], which reported reduced HRR and exercise capacity in patients with metabolic syndrome. However, the factors of the metabolic syndrome affecting HRR including diabetes were not analyzed, and thus, an additional study on this topic is necessary.

CAC score is a method that can easily detect subclinical atherosclerosis using CTA, and CAC score allows to predict the risk for developing CVD [23, 24]. In this study, the CAC score was significantly higher in Groups 3 and 2 than in Group 1, and this result is similar to previous studies, which reported that a higher number of components for metabolic syndrome indicated higher CAC and an increased risk of CVD [2, 25].

In addition, exercise duration, which reflects exercise capacity on ETT, and CAC showed a negative correlation, indicating an association between reduced exercise duration and CAC. Furthermore, reduced exercise duration could predict the risk for CAD. However, the correlation between CAC and exercise duration based on the components for metabolic syndrome was different for each coronary artery.

Increased left ventricular mass and dysfunction are associated with the development of CAD, stroke, and heart failure [26–28]. In the echocardiography conducted in this study, Group 3 had a significantly greater ventricular end-diastolic diameter, and IVS and PW thickness. Peak late diastolic transmitral flow, an indicator of the diastolic function of the left ventricle, was also high in Group 3, while the E/A ratio was small. This agrees with the results of previous studies [4–6], which reported deterioration in cardiac structure and function in patients with metabolic syndrome, indicating the involvement of metabolic syndrome in ventricular remodeling and dysfunction that affects the heart. However, there was no difference in the left ventricular ejection fraction, an indicator of the systolic function of the left ventricle between the 3 groups, and all showed a mean value greater than 60%. A previous study reported that metabolic syndrome reduced the diastolic function of the left ventricle but had no effect on the systolic function, [29]. Masugata et al. [5] reported a decline in the diastolic function of the left ventricle in patients with metabolic syndrome, but no change in the left ventricular ejection fraction, an indicator of systolic function. These are in line with the results of this study. Based on such findings, metabolic syndrome, a composite of a series of CVD risk factors, can affect the left ventricular structure resulting in a decline in the diastolic function but does not seem to affect the systolic function directly, like hypertension or diabetes.

Furthermore, peak early and late diastolic transmitral flows and E/A ratio showed a statistically significant correlation with METs during maximal exercise, exercise duration, and HRR, indicating that the decline in left ventricle function seen on echocardiography resulted in an actual decline in exercise capacity. In addition, the peak late diastolic transmitral flow showed a statistically significant positive correlation with the calcium score of each coronary artery except the right coronary artery, total calcium score, and calcium volume. Peak late diastolic transmitral flow indicates the diastolic function of the left ventricle on echocardiography; thus an increased flow can help predict the possibility of progression to CAC.

The limitations of this study are as follows: first, the study subjects consisted of people who visited the hospital for health examination without any specific symptoms. Therefore, their lifestyle and prevalence of chronic diseases might differ from the general population. In fact, the prevalence of hypertension, diabetes, and hyperlipidemia based on the medical history of the study subjects were higher than in the general Korean population. Second, exercise capacity measurement using symptom-limited ETT did not directly assess the maximum oxygen uptake (VO2max). Since indirectly estimated METs during maximal exercise were measured, the exact exercise capacity might not have been reflected. In the future, a long-term prospective study consisting of a larger number of study subjects from the general population is necessary.

## 5. Conclusion

Patients with metabolic syndrome showed a decline in exercise capacity compared to patients without component or those with 1, 2 components. The calcification of the coronary artery was higher and the cardiac structure and functions were more deteriorated. These findings suggest that metabolic syndrome reduces the exercise capacity and contributes to calcification of the coronary arteries, increasing the risk of cardiovascular disease, and deteriorating the cardiac structure and function. Therefore, active monitoring, management, and intervention are necessary to prevent the development of heart diseases in patients with metabolic syndrome, in addition to efforts for early detection.

## Figures and Tables

**Table 1:** Definition of the metabolic syndrome according to ATP III criteria.

	Original ATP III	ATP III-BMI 25
Visceral obesity	≥102 for men	
WC (cm)	≥88 for women	
BMI (kg/m2)		≥25
High BP (mmHg)	≥130/85	≥130/85
High TG	≥150	≥150
Low HDL-	<40 for men	<40 for men
C (mg/dL)	<50 for women	<50 for women
High glucose (mg/dL)	≥110	≥110

WC, waist circumference; BMI, body mass index; BP, blood pressure; TG, triglyceride; HDL-C, high density lipoprotein cholesterol.

**Table 2:** Percentage of subjects that have each components of metabolic syndrome.

	Group 1	Group 2	Group 3	All subjects
	(%)	(%)	(%)	(%)
BMI (≥25kg/m2)	0	139 (43.3)	132 (85.7)	271 (48.0)
High BP (≥130/85mmHg)	0	99 (30.8)	64 (41.6)	86 (15.2)
High TG ( ≥150 mg/dL)	0	55 (17.1)	81 (52.6)	136 (24.2)
Low HDL-C				
(<40mg/dL for men	0	83 (25.9)	73 (47.4)	164 (29.0)
<50mg/dL for women)				
High Fasting plasma glucose (≥110mg/dL)	0	27 (8.4)	65 (42.2)	92 (16.3)
All components	0	0	9 (5.8)	9 (1.6)

BMI, body mass index; BP, blood pressure; TG, triglyceride; HDL-C, high density lipoprotein cholesterol.

**Table 3:** General characteristics and laboratory findings of the subjects.

	Group 1	Group 2	Group 3
	(n=90)	(n=321)	(n=154)
Male, n (%)	43 (47.8)	190 (59.2)	107 (69.5)
Age (years)	57.7 ± 7.8	60.2 ± 8.9	59.1 ± 12
Height (cm)	163.6 ± 7.9	163.3 ± 8.8	165.7 ± 9.1
Weight (kg)	59.1 ± 7.9	66.3 ± 10.9^a)^	75.3 ± 11.7^a),b)^
BMI (kg/m2)	22 ± 1.9	24.8 ± 3.1^a)^	27.3 ± 2.9^a),b)^
HbA1c (%)	5.3 ± 0.4	5.7 ± 0.9^a)^	6.5 ± 1.3^a),b)^
Fasting plasma glucose (mg/dL)	84 ± 9.7	92.4 ± 25.1^a)^	112.7 ± 34.1^a),b)^
Total cholesterol (mg/dL)	200.3 ± 27.5	205.3 ± 41.6	195.9 ± 40.7
Triglyceride (mg/dL)	73.5 ± 31.9	111.2 ± 85.5^a)^	180 ± 129.5^a),b)^
HDL-C (mg/dL)	59.9 ± 12.2	54 ± 13.1^a)^	47.1 ± 9.8^a),b)^
LDL-C (mg/dL)	114.6 ± 27.8	120.7 ± 34.6	118 ± 57.3

Values are presented as mean ± standard deviation.

BMI: body mass index; HDL-C: high density lipoprotein cholesterol; LDL-C: low density lipoprotein cholesterol.

a) p<0.05 compared with group 1.

b) p<0.05 compared with group 2.

**Table 4:** Results of exercise tolerance test, coronary artery calcium score, and echocardiography according to the groups.

	Group 1	Group 2	Group 3
Exercise tolerance test			
Exercise duration (sec)	543.1 ± 113.2	528.2 ± 117.2	511.5 ± 127.7^a)^
METs	11.5 ± 2	11.3 ± 6.9	10.6 ± 2.4^a)^
HRR (bpm)	31.6 ± 12.2	30 ± 11.4	25.4 ± 9.6^a),b)^
Agatston score			
LMA	2.9 ± 22.7	1.6 ± 9.1	8.5 ± 36.4
LADA	8.2 ± 35.9	16.2 ± 54.3	48.1 ± 161^a),b)^
LCXA	0.6 ± 2.9	5.5 ± 25.8^a)^	14.4 ± 66^a)^
RCA	0.6 ± 3.5	10.7 ± 46.9^a)^	40.4 ± 176.4^a)^
Total	12.8 ± 45.1	34.3 ± 108.6^a)^	111.4 ± 379.7^a),b)^
Calcium volume	10.2 ± 36	27.6 ± 86.5^a)^	89.8 ± 298.9^a),b)^
Echocardiography			
LVEDD (mm)	48.1 ± 6.3	49.2 ± 4.4	49.6 ± 4.2^a)^
LVESD (mm)	31.3 ± 3.4	34.6 ± 5.7	31.4 ± 3.7
IVS (mm)	8.7 ± 1.3	9.6 ± 2.7^a)^	10.1 ± 1.4^a),b)^
PW (mm)	8.7 ± 1.1	9.3 ± 1.2^a)^	9.7 ± 1.1^a),b)^
LVEF (%)	64.7 ± 5.5	65.9 ± 6.3	65.9 ± 6.4
E (cm/sec)	54.5 ± 13.3	54.2 ± 13.5	54.4 ± 13
A (cm/sec)	55.4 ± 11.4	62.7 ± 14.5^a)^	65.4 ± 14.1^a)^
E/A	1 ± 0.3	0.9 ± 0.3^a)^	0.9 ± 0.3^a)^

Values are presented as mean ± standard deviation.

SBP: systolic blood pressure; DBP: diastolic blood pressure; HR: heart rate; METs: metabolic equivalent tasks; HRR: heart rate recovery; LMA: left main artery; LADA: left anterior descending artery; LCXA: left circumflex artery; RCA: right coronary artery; LVEDD: left ventricular end-diastolic diameter; LVESD: left ventricular end-systolic diameter; IVS: interventricular septum thickness; PW: posterior wall thickness; LVEF: left ventricular ejection fraction; E: peak early diastolic transmitral flow; A: peak late diastolic transmitral flow; E/A: the ratio of E to A.

a) p<0.05 compared with group 1.

b) p<0.05 compared with group 2.

**Table 5:** Results of multivariate logistic regression analysis.

	Model 1			Model 2		
	aOR	95% CI		aOR	95% CI	
		Lower	Upper		Lower	Upper
Exercise tolerance test						
Exercise duration (sec)	1.000	0.998	1.002	0.997	0.995	1.000
METs	1.023	0.935	1.119	0.880	0.770	1.006
HRR (bpm)	0.991	0.971	1.012	0.940	0.913	0.969
Agatston score						
Total	1.679	0.969	2.910	3.003	1.636	5.513
Calcium volume	1.679	0.969	2.910	3.003	1.636	5.513
Echocardiography						
LVEF (%)	1.029	0.989	1.070	1.032	0.986	1.080
E/A	0.277	0.110	0.699	0.108	0.035	0.336

Model 1: group 2 vs group 1.

Model 2: group 3 vs group 1.

METs: metabolic equivalent tasks; HRR: heart rate recovery; LVEF: left ventricular ejection fraction; E: peak early diastolic transmitral flow; A: peak late diastolic transmitral flow; E/A: the ratio of E to A.

OR: adds ratios.

**Table 6:** Correlation analysis between the exercise tolerance test and Agatston score.

	METs	Exercise duration	HRR
Agatston score			
LMA	-0.04	-0.07	-0.06
LADA	-0.04	-0.07	-0.05
LCXA	-0.03	-0.07	0
RCA	-0.04	-0.1^*∗*^	-0.06
Total	-0.05	-0.1^*∗*^	-0.05
Calcium volume	-0.05	-0.1^*∗*^	-0.05

Values are Pearson correlation coefficients.

METs: metabolic equivalent tasks; HRR: heart rate recovery; LMA: left main artery; LADA: left anterior descending artery; LCXA: left circumflex artery; RCA: right coronary artery.

^*∗*^p <0.05 by Pearson correlation analysis.

**Table 7:** Correlation analysis between exercise tolerance test and echocardiographic parameters.

	METs	Exercise duration	HRR
LVEDD (mm)	0.02	0.04	0
LVESD (mm)	0.02	0.03	-0.03
IVS (mm)	-0.05	-0.08	-0.11^*∗*^
PW (mm)	0.02	-0.02	-0.16^*∗*^
LVEF (%)	-0.05	-0.02	0.04
E (cm/sec)	-0.1^*∗*^	-0.04	0.09^*∗*^
A (cm/sec)	-0.17^*∗*^	-0.27^*∗*^	-0.06
E/A	0.05	0.18^*∗*^	0.12^*∗*^

Values are Pearson correlation coefficients.

METs: metabolic equivalent tasks; HRR: heart rate recovery; LVEDD: left ventricular end-diastolic diameter; LVESD: left ventricular end-systolic diameter; IVS: interventricular septum thickness; PW: posterior wall thickness; LVEF: left ventricular ejection fraction; E: peak early diastolic transmitral flow; A: peak late diastolic transmitral flow; E/A: the ratio of E to A.

^*∗*^p <0.05 by Pearson correlation analysis.

**Table 8:** Correlation analysis between Agatston score and echocardiographic parameters.

	LMA	LADA	LCXA	RCA	Total	Calcium volume
LVEDD (mm)	0	0.03	-0.03	-0.01	-0.01	-0.01
LVESD (mm)	-0.01	-0.01	-0.01	-0.01	-0.01	-0.01
IVS (mm)	0.03	-0.12^*∗*^	0.06	0.04	0.08	-0.08
PW (mm)	0.04	0.09^*∗*^	0.02	0.05	0.07	0.07
LVEF (%)	0.05	-0.01	0.02	-0.06	-0.02	0.02
E (cm/sec)	0.06	0.01	0.06	0.03	0.04	0.04
A (cm/sec)	0.08^*∗*^	0.1^*∗*^	0.08^*∗*^	0.08	0.1^*∗*^	0.1^*∗*^
E/A	-0.03	-0.08	-0.05	-0.05	-0.07	-0.07

Values are Pearson correlation coefficients.

LMA: left main artery; LADA: left anterior descending artery; LCXA: left circumflex artery; RCA: right coronary artery; LVEDD: left ventricular end-diastolic diameter; LVESD: left ventricular end-systolic diameter; IVS: interventricular septum thickness; PW: posterior wall thickness; LVEF: left ventricular ejection fraction; E: peak early diastolic transmitral flow; A: peak late diastolic transmitral flow; E/A: the ratio of E to A.

^*∗*^p <0.05 by Pearson correlation analysis.

## Data Availability

The data used to support the findings of this study are included within the article.
